# Predicting Job Burnout Among Female Nurses in China With Machine Learning and Shapley Additive Explanations

**DOI:** 10.1155/jonm/2572646

**Published:** 2025-12-28

**Authors:** Xue Hu, Chong Liu, Xiaoshi Yang

**Affiliations:** ^1^ College of Health Management, China Medical University, Shenyang, 110122, Liaoning, China, cmu.edu.tw; ^2^ Shengjing Hospital of China Medical University, Shenyang, 110022, Liaoning, China, cmu.edu.cn

## Abstract

Job burnout among nurses is prevalent globally, particularly in China. However, few studies have been conducted on reliable tools for building predictive models. This cross‐sectional study was conducted in four cities of Liaoning Province in China during the period from January to April 2022 by utilizing a self‐administered smartphone questionnaire protocol, yielding 1400 responses from female nurses. We applied the least absolute shrinkage and selection operator (LASSO) and Boruta to identify the common predictors of job burnout. We then adopted and optimized three highly applicable machine learning (ML) algorithms—K‐nearest neighbor (KNN), EXtreme Gradient Boosting (XGBoost), and random forest (RF)—to predict job burnout among female nurses. The values of area under curve (AUC) of KNN, RF, and XGBoost ML models were 0.85–0.95, with XGBoost performing best (AUC = 0.939). In addition, Shapley additive explanations (SHAP) were used to show the contribution of each predictor to the predicted outcomes. The result confirmed the role of consistency, perceived stress, and physical fatigue as key protective factors, and consistency exhibited interactions with perceived stress, organizational support, psychological detachment, and sense of control to nurses’ job burnout. This helps identify nurses at risk of job burnout and provide targeted strategy to alleviate nurses’ job burnout.

## 1. Introduction

Job burnout among nurses has become one of the most prevalent global public health issues [1]. Job burnout is a syndrome resulting from chronic workspace stress that has not yet been successfully managed. Nurses, as frontline healthcare workers, are directly involved in patient treatment and daily healthcare [2–4], constituting the largest proportion of healthcare professionals in health systems. Day‐ and night‐shift patterns, long working hours, and daily exposure to job stressors such as patient distress, negativity, helplessness, and other workplace‐related conflicts make nurses more susceptible to job burnout [5–8]. The prevalence of job burnout among nurses significantly distracts from achieving the sustainable development goals (SDGs), which aim to ensure a healthy life, promote well‐being for everyone at all ages, and so on [9–11]. Therefore, addressing job burnout is critical for advancing global health and sustainability objectives related to SDGs.

Studies have shown that the global prevalence of job burnout is approximately 30.0% [12–14]. However, in China, research has revealed that 43.5%–62.0% of Chinese nurses suffer from job burnout [15]. In comparison, the incidence of job burnout is higher among Chinese nurses. High levels of job burnout have been shown to affect nurses’ mental health (depression, anxiety, suicide, etc.) [16–18] and to be correlated with an increased risk of nurses suffering from headaches, hypertension, cardiovascular disease, and musculoskeletal pain [19–21]. This affects the quality of care they provide to patients, increasing the likelihood of medical errors, and resulting in high levels of job withdrawal [22–25]. Therefore, job burnout among frontline nurses in China should be paid special attention. In addition, female nurses remain the mainstay of the nursing profession and the core force in nursing services, and their occupational health is directly linked to the stability of the medical system in China [26]. Multiple studies have confirmed that female nurses have a higher risk of suffering from emotional exhaustion than male nurses (*p* < 0.05) [27–29]. Based on the Conservation of Resources Theory [30] and the Job Demands‐Resources Model [31], female nurses are required to take on multiple roles simultaneously, including high‐intensity clinical work as well as family caregiving and nurturing responsibilities. They have long faced an imbalance between job resources (e.g., organizational support, shift flexibility) and demands, making them more prone to burnout [32]. Consequently, our research mainly concerned on the job burnout among female nurses.

Research on the factors influencing nurses’ job burnout has focused on personal characteristics and work‐related features. The most frequently discussed of these are work‐related influences, such as high workloads, work patterns, shift systems, and workplace violence, all of which are closely associated with nurses’ job burnout [33–35]. A favorable working climate, positive working relationships, and social support protect against job burnout [36–38]. Many studies have shown that demographic characteristics such as age, education, and marital status are associated with job burnout [39, 40]. In addition, mental health (depression, anxiety, insomnia, etc.) [23, 41] and health behavior‐related behaviors (smoking, drinking, etc.) [19] are associated with nurses’ job burnout. However, most studies have focused on related factors, and there is little research on reliable tools for building predictive models.

In recent years, with the development of statistical theory and computer technology, machine learning (ML), including random forests (RF), K‐nearest neighbors (KNN), and Extreme Gradient Boosting (XGBoost), has been widely used for disease diagnosis, hazard identification, and health decision‐making [42–45]. Compared to traditional methods, ML algorithms have many advantages in detecting nonlinear associations, complex interactions, and efficient processing of big data [46, 47]. Existing studies have made valuable contributions to predicting nurses’ job burnout through ML. For instance, one study developed an early screening tool for burnout using a convolutional neural network (CNN) [48]; Rocha et al. [49] employed K‐means clustering and RF algorithms to identify protective factors for burnout among oncology nurses; Zeng et al. [50] employed ML and Shapley Additive exPlanations (SHAP) to identify key factors influencing job burnout among nurses in Shandong Province, drawing on the job demands‐resources model. These studies have laid a solid foundation, and building on them, our research incorporated these relevant dimensions including demographic characteristics, internal personal features, and external work‐related factors into the analytical models. By combining ML with SHAP methods, we construct an interpretable predictive framework for job burnout among female nurses in Liaoning Province to clarify feature contributions and explore the key predictors influencing job burnout.

Feature screening is a critical step in the training set [51]. The Boruta algorithm is a global feature selection method based on RF, which is insensitive to noisy data and performs well when dealing with complex datasets [52]. LASSO is a popular embedded feature selection method that tends to compress unimportant coefficients to zero by adding an L1 regularization term to the loss function [53]. In our study, we employed Boruta and LASSO‐based feature selection methods to identify the common predictors of job burnout. These approaches help to reduce the number of features in the model and solve the problem of multicollinearity by automatically selecting the most useful features for prediction, which have been widely used [54–56].

Therefore, this study aimed to (1) screen features by using Boruta and LASSO and (2) construct interpretable predictive models by applying ML and SHAP, providing critical tools to identify nurses at high burnout risk and enabling interventions targeting core measures to alleviate nurses’ job burnout.

## 2. Materials and Methods

### 2.1. Study Design and Participants

This study is a cross‐sectional design, implemented from four cities of Liaoning Province in China during the period from January to April 2022. First, public hospitals in four Liaoning cities (Shenyang, Fushun, Fuxin, Liaoyang) were selected. Then, clinical departments within these eight chosen hospitals were sampled, with approximately 30% of nurses invited to participate. Data collection employed a self‐administered smartphone questionnaire (via Wenjuanxing), assessing job burnout and related factors. The first section of the questionnaire clearly outlined the study’s purpose, estimated completion time, anonymity principle, and that data would be used solely for academic research and protected by law. Participants were required to click the “Agree” button to proceed to the subsequent questions. This design ensured that participants made informed and voluntary decisions after fully understanding the study details and their rights. All the participants were well informed of the purposes and contents of this study. Finally, 1400 valid questionnaires were actually collected with an affective rate of 93.3% in this study. Our research team previously utilized this dataset to publish an article about depressive symptoms among nurses [57]. Differently, in the current study, we concentrated on investigating the influencing factors of job burnout among female nurses.

### 2.2. Participants

The study included 1400 clinical female nurses recruited from eight general hospitals in four northern Liaoning cities (Shenyang, Fushun, Fuxin, Liaoyang) via proportional random sampling. These nurses were at least 18 years old, capable of completing an online Wenjuanxing questionnaire, voluntarily agreed to participate in the survey, and did not have diagnoses or treatment histories for severe mental illnesses (like bipolar or psychotic disorders). Data from these eligible individuals formed the study sample.

The sample size was calculated based on the following formula:

(1) N=Z2P1−Pd2.

Based on an estimated 30% prevalence rate for job burnout among nurses and the basis (Π = 30%) for sample size estimation, the allowable error “*d*” was set at 3.5% to ensure accuracy; for a 95% confidence interval, *α* = 0.01 and *Z* = 2.58. Considering a 20% follow‐up loss rate, the estimated sample size was 1369. Ultimately, 1500 questionnaires were collected. After excluding invalid ones and male participants, 1400 valid responses were obtained, yielding a 93.3% response rate.

### 2.3. Variables

#### 2.3.1. Outcome Variable

This study used the Chinese version of the Maslach Burnout Inventory‐General Survey to measure nurses’ job burnout, which has been previously validated among Chinese healthcare professionals [58, 59]. It comprises 16 items that cover three dimensions: exhaustion, cynicism, and professional efficacy. The classification of burnout was set as follows: no job burnout (0–1.49), mild job burnout (1.50–3.49), and severe job burnout (3.50–6) [54, 55]. Participants with mild or severe burnout were defined as “job burnout.” The Cronbach *α* coefficient of this scale was 0.914.

(2) Job burnout=0.40.30.3∗Exhaustion+∗Cynicism+∗Professional Efficacy.

#### 2.3.2. Predictive Variables

This study categorized predictors of nurses’ job burnout as demographic characteristics, internal personal features, and external work‐related factors. The demographic characteristics included age, marital status, income, and chronic diseases. Internal personal features included recovery experience, resilience, depression, empathy, anxiety, sleep quality, perceived stress, fatigue, coping styles, and career identity. External work‐related factors include workweek, verbal abuse, physical violence, and organizational support.

The recovery experience is composed of four dimensions: psychological detachment, relaxation, mastery, and control. Three dimensions are integrated into fatigue: physical, affective, and cognitive. Three dimensions are involved in coping style: problem‐focused, emotion‐focused, and avoidant coping. Career identity consists of seven dimensions: grasp, consistency, significance, self‐efficacy, self‐decision, organizational influence, and individual influence. Empathy has three dimensions: perspective adoption, compassionate care, and walking in patient’s shoes. The remaining features contain only one dimension. Detailed descriptions of scales can be found in Scale S1 of Supporting Information.

We took into consideration all the dimensions in the analytic models to thoroughly explore the factors affecting job burnout among female nurses. Thereby, 33 features were yielded, covering demographic characteristics, internal personal attributes, and external work‐related elements.

### 2.4. Data Analysis

This study used SPSS 27.0 and Python version 3.12.3 for data analysis. All the ML methods used the scikit‐learn package. Statistically significant was defined as *p* < 0.05.

First, based on the proportion of 8 : 2, the data were randomly split into a training set (*n* = 1120) and validation set (*n* = 280). The demographic characteristics of the female nurses were described using descriptive statistics. Categorical variables were described as percentages and compared using the chi‐squared test. We calculated the correlations among all dimensions using Pearson’s correlation analysis. The variables with statistical significance were included in the next step of feature screening.

We used LASSO and Boruta to screen features of job burnout in the training set. We then adopted highly applicable ML algorithms, including KNN, XGBoost, and RF. The hyperparameters were optimized using a grid search method with cross‐validation protocols to obtain the best model prediction performance during the training process. The performance of the models was assessed by relying on accuracy, precision, recall, *F*1 score, and AUC obtained from the receiver operating characteristic (ROC) curve on the test set.

Finally, to better interpret and understand the contribution of each factor in the model to the prediction results, we employed the powerful SHAP technique to visualize the prediction models with the best performance (summary plot, dependence plot, etc.).

**Figure 1:**
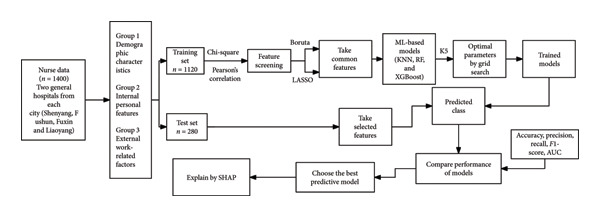
Flowchart of this study.

The detailed flowchart of the research process is shown in Figure 1.

### 2.5. Ethical Considerations

This study complied with the Declaration of Helsinki (1989) and obtained the consent of the Ethics Committee of China Medical University (Grant ID: 2020048). Written informed consent was obtained from all the participants.

## 3. Results

### 3.1. Demographic and Work‐Related Characteristics

The prevalence of job burnout among the 1400 female nurses participating in the study was 72.00%, with detailed characteristics of these participants presented in Table 1. Approximately 45.50% of nurses were older than 35 years; most were married (78.43%) and had a monthly household income less than 6000 RMB (73.21%). Nearly a quarter (23.64%) of the female nurses reported a chronic illness, and a similar proportion (23.14%) had employment exceeding 50 h per week. High rates of exposure to verbal violence (87.36%) were observed, though physical violence was less common (25.71%). In both the training and test sets, there was a significant difference in prevalence based on age, monthly income, workweek, verbal violence, and physical violence. Therefore, we input these five feature variables into the next feature filter.

**Table 1:** Analysis of demographic and work‐related characteristics among the female nurses.

Demographics features	Training set			Validation set		
	Job burnout (%)	No job burnout (%)	*p* value^∗^	Job burnout (%)	No job burnout (%)	*p* value^∗^
Total	802 (71.61)	318 (28.39)		206 (73.57)	74 (26.43)	
Age (years)			**0.015** ^∗^			**0.046** ^∗^
≤ 35	450 (56.10)	153 (48.10)		125 (60.70)	35 (47.30)	
> 35	352 (43.90)	165 (51.90)		81 (39.30)	39 (52.70)	
Marital status			0.945			0.113
Married	632 (78.80)	250 (78.60)		154 (74.80)	62 (83.80)	
Others	170 (21.20)	68 (21.40)		52 (25.20)	12 (16.20)	
Income			**0.001** ^∗∗^			**0.003** ^∗∗^
≤ 6000	632 (78.80)	190 (59.70)		159 (77.20)	44 (59.50)	
> 6000	170 (21.20)	128 (40.30)		47 (22.80)	30 (40.50)	
Workweek			**0.001** ^∗∗^			**0.037** ^∗^
≤ 50 h	598 (74.60)	273 (85.80)		144 (69.90)	61 (82.40)	
> 50 h	204 (25.40)	45 (14.20)		62 (30.10)	13 (17.60)	
Verbal abuse			**0.001** ^∗∗^			**0.001** ^∗∗^
Never	71 (08.85)	68 (21.40)		25 (12.10)	13 (17.60)	
Seldom	392 (48.88)	178 (56.00)		91 (44.20)	47 (63.50)	
Usually	339 (42.27)	72 (22.60)		90 (43.70)	14 (18.90)	
Physical violence			**0.001** ^∗∗^			**0.003** ^∗∗^
Yes	225 (28.10)	56 (17.60)		68 (33.00)	11 (14.90)	
No	577 (71.90)	262 (82.40)		138 (67.00)	63 (85.10)	
Chronic disease			0.062			0.822
Yes	201 (25.10)	63 (19.80)		50 (24.30)	17 (23.00)	
No	601 (74.90)	255 (80.20)		156 (75.70)	57 (77.00)	

*Note:* ^∗^Chi‐squared test for all demographics characteristics. ^∗^ *p* < 0.05, ^∗∗^ *p* < 0.01. Bold values indicate significant differences.

### 3.2. Internal and External Features

**Figure 2:**
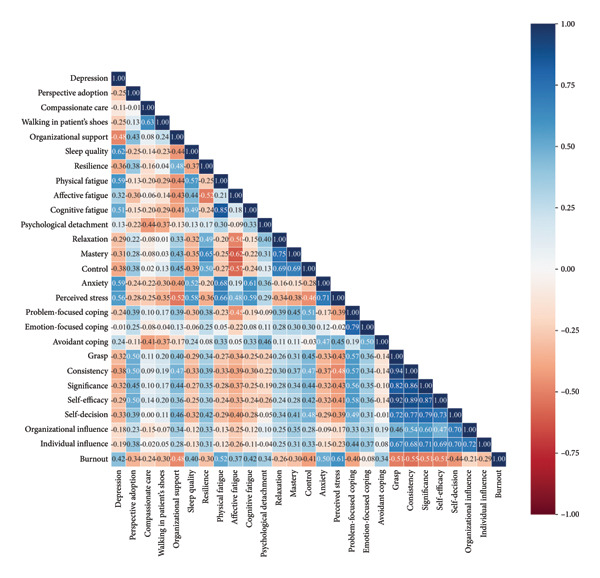
The heatmap of Pearson’s correlation.

A heatmap of Pearson’s correlation was employed to illustrate the correlations among internal and external features and job burnout in Figure 2. As shown in the graph, it is clear that job burnout was highly and negatively correlated with the seven dimensions of career identity. All the dimensions of recovery experience, empathy, avoidant coping, and resilience were negatively correlated with job burnout. Depression, sleep quality, fatigue, anxiety, and perceived stress are positively associated with job burnout. Regarding the three dimensions in the Brief‐COPE scale, problem‐focused coping was negatively correlated with nurses’ job burnout, while avoidant coping was positively correlated with it. The correlation coefficient of emotion‐focused coping was small and not statistically significant (*p* > 0.05); therefore, it was excluded. Finally, we placed the remaining 25 feature variables for the next feature screening.

### 3.3. Feature Screening Using LASSO and Boruta

**Figure 3:**
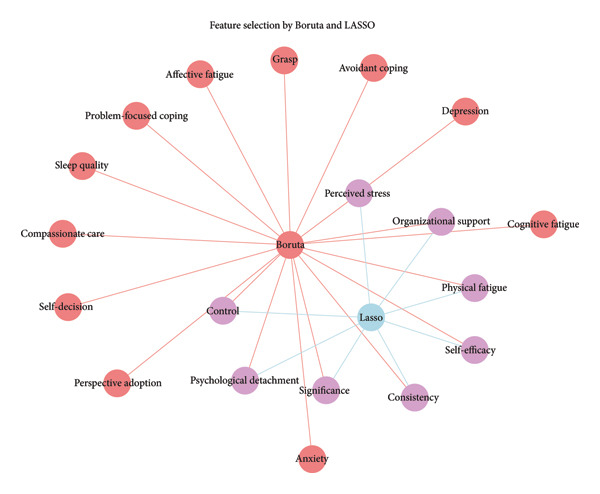
Common predictors between Boruta and LASSO.

Figure 3 shows the results of feature screening using LASSO and Boruta. While Boruta identified 19 potential predictors (marked in orange in Figure 3), LASSO identified a smaller subset of predictors, consisting of eight features (marked in purple in Figure 3). Finally, eight common features (organizational support, physical fatigue, psychological detachment, control, perceived stress, consistency, significance, and self‐efficacy) were included as risk factors to develop an ML model for predicting nurses’ job burnout in Figure 3. The LASSO path and alpha value diagram are provided in Figure S1 of Supporting Information.

### 3.4. Performance Comparison of ML‐Based Models

**Figure 4:**
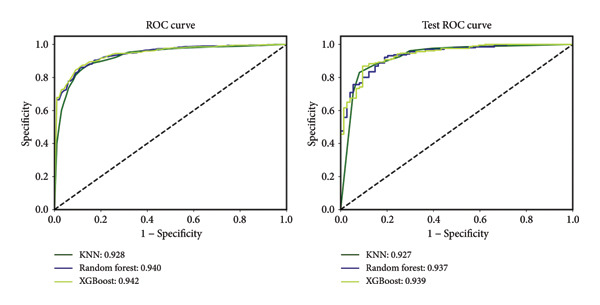
The ROC curves of the three ML‐based models on the training and test sets.

Figure 4 shows a comparison of the ROC curves of the three ML‐based models for the training and test sets. It is clear that the values of AUC of KNN, RF, and XGBoost ML models are approximately 0.85–0.95, with good discriminative ability overall, in which XGBoost performs the best. Moreover, Scikit‐learn GridSearchCV with cross‐validation was used to adjust the parameter values to obtain the best model prediction performance during the training process. The results of tuned hyperparameters are shown in Table S1 of Supporting Information, and the model performance after parameter adjustment is presented in Table 2. In the test set, the XGBoost model achieved a predictive accuracy of 0.889, a precision of 0.907, a recall of 0.947, an *F*1 score of 0.926, and an AUC of 0.939, which were better than those of the other two models.

**Table 2:** The results of models for the job burnout on the training and test sets. The model performances after parameter adjustment on the training and test.

Data sets	Model	Accuracy	Precision	Recall	*F*1 score	AUC
Training set	KNN	0.87 ± 0.03	0.903 ± 0.04	0.918 ± 0.03	0.91 ± 0.02	0.93 ± 0.03
	RF	0.87 ± 0.03	0.893 ± 0.04	0.93 ± 0.02	0.911 ± 0.02	0.943 ± 0.02
	XGBoost	0.867 ± 0.03	0.888 ± 0.04	0.932 ± 0.03	0.909 ± 0.02	0.945 ± 0.02

Test set	KNN	0.879	0.91	0.927	0.918	0.927
	RF	0.879	0.898	0.942	0.919	0.937
	XGBoost	0.889	0.907	0.947	0.926	0.939

### 3.5. SHAP Interpretation Predictors

Figure 5(a) presents a heatmap generated by the XGBoost model. Varied color gradients illustrate both the direction and magnitude of the influence exerted by different feature value levels on the model output. Warm colors correspond to positive SHAP values, which exert a promoting effect on the prediction result, while cool colors represent negative SHAP values that exert an inhibitory effect on the prediction result. Deeper color intensity indicates larger average absolute SHAP values and thus stronger predictive contributions. Figure 5(b) provides a standard bar chart of the average absolute SHAP values for all predictors. The summary plot (Figure 5(c)) illustrates the direction of feature values’ impacts. The presentation of three types of plots helps rule out potential biases associated with single analytical approaches, thereby ensuring the robustness of the analytical results.

Figure 5SHAP analysis results of the XGBoost model.

**Figure:**
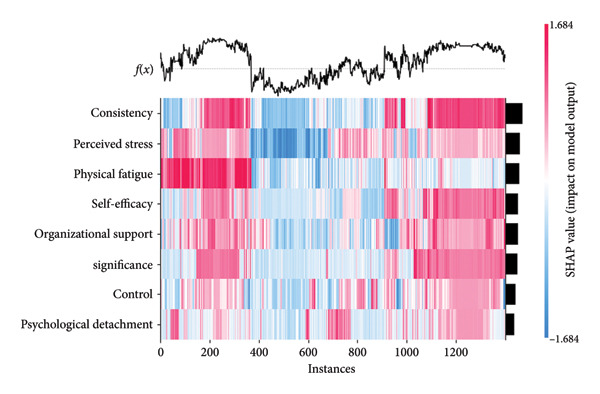
(a)

**Figure:**
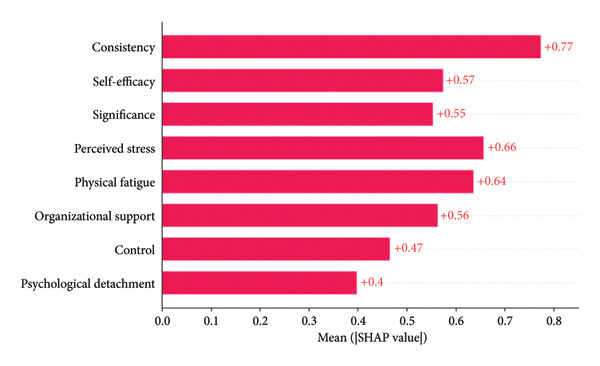
(b)

**Figure:**
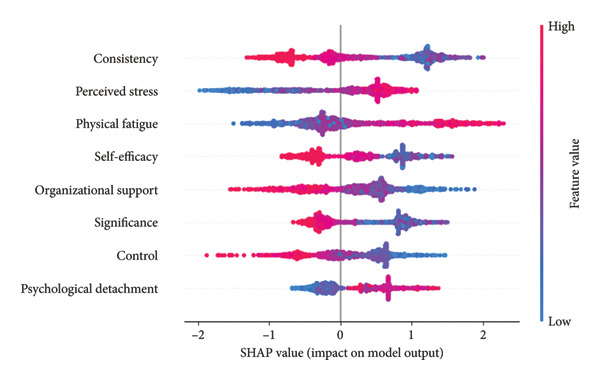
(c)

In the heatmap with 1400 samples, the cool‐color blocks in the row corresponding to consistency had the widest coverage and the highest intensity, indicating that its protective effect against nurses’ job burnout far exceeded that of other variables, making it the strongest protective factor in the model. This is consistent with the ranking of average absolute SHAP values in Figure 5(b) (average |SHAP| = 0.77), and Figure 5(c) also shows that consistency is negatively correlated with job burnout.

Perceived stress and physical fatigue corresponded to warm‐color blocks with the most intense color and the widest coverage in the heatmap, identifying them as core risk factors that promote nurses’ job burnout. This conclusion aligns with the ranking of average absolute SHAP values in Figure 5(b) (average |SHAP| of perceived stress = 0.66; average |SHAP| of physical fatigue = 0.64), and Figure 5(c) also reveals a positive correlation between these two factors and job burnout.

Self‐efficacy (average |SHAP| = 0.57), organizational support (average |SHAP| = 0.56), and significance (average |SHAP| = 0.55) were all negatively correlated with job burnout. Although the intensity of their cool‐color blocks in the heatmap was lower than that of consistency, they covered a wider range of samples (e.g., self‐efficacy), classifying them as important protective factors against nurses’ job burnout. This is fully consistent with their ranking in Figure 5(b) and the direction of impact shown in Figure 5(c).

For control (average |SHAP| = 0.47), the cool‐color blocks in the heatmap had weak intensity and covered few samples, resulting in a limited regulatory effect on nurses’ job burnout. As for psychological detachment (average |SHAP| = 0.40), its pale warm‐color blocks covered an extremely small number of samples, and its average absolute SHAP value was the lowest. Its promoting effect on job burnout was far weaker than that of perceived stress and physical fatigue.

**Figure 6:**
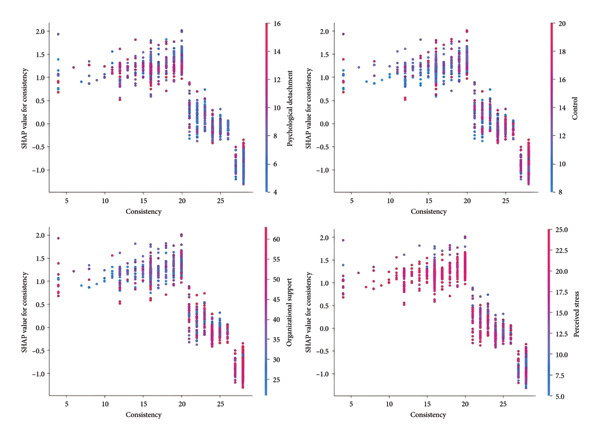
The dependence plots between consistency with psychological detachment, control, organizational support, and perceived stress.

As illustrated in the dependence plot (Figure 6), the sense of consistency, which was previously identified as the strongest protective factor against nurses’ job burnout, exhibited interactions with four key variables, perceived stress, organizational support, psychological detachment, and sense of control. It shows that among nurses with high perceived stress, a low sense of consistency strongly promotes nurses’ job burnout. For nurses receiving ample organizational support, a high sense of consistency was associated with a significantly lower predicted risk of job burnout. Nurses with both high consistency and high sense of control showed lower burnout levels. For nurses with moderate psychological detachment, a low sense of consistency exerted a protective effect.

## 4. Discussion

Our study contributes to the existing literature in several ways. First, regarding the issue of feature selection, we adopted a dual feature selection strategy of Boruta and LASSO to screen for common predictors of job burnout. This design significantly enhanced the robustness of key factors and avoided the false positive risk that may be caused by a single feature selection method. All models exhibited good performance, and among them, the XGBoost model performed best. This provides a more reliable methodological reference for the accurate prediction of nurses’ job burnout in subsequent studies.

The core contribution of this study is identifying the key risk and protective factors of nurses’ job burnout as well as their complex interactions through ML and SHAP and designing more targeted and predictive situational intervention measures, which are expected to more effectively reduce the level of nurses’ job burnout. Currently, burnout intervention measures in nursing management often rely on generalized universal approaches (such as self‐care activities and mindfulness practices) and lack specificity to the needs of individuals or groups [60, 61]. However, based on the results of this study, we propose a stratified and targeted intervention framework for future nursing management.

It shows that the consistency dimension of career identity is the strongest protective factor against nurses’ job burnout, which is consistent with the conclusion in previous studies that higher career identity is associated with lower burnout levels [62, 63]. Moreover, this study further clarifies that consistency plays a core protective role within career identity. In addition, we found that the other two dimensions of career identity (sense of significance and self‐efficacy) also have important impacts on burnout, and consistency exhibited interactions with perceived stress, organizational support, psychological detachment, and sense of control with respect to nurses’ job burnout. For nurses with low consistency levels, interventions can not only integrate the other two dimensions of career identity (sense of significance and self‐efficacy) to implement a career meaning reconstruction program. This program includes activities such as organizing nursing achievement seminars and mapping personalized career trajectories, which are designed to promote the alignment between their professional values and daily work experiences. Interventions can also establish a rapid problem response mechanism: for cognitive conflicts encountered in work (e.g., the contradiction between nursing operation standards and patient needs), head nurses take the lead in providing solutions within 24 h to improve the sense of control and prevent the job burnout. This finding also implies the need to optimize the sense of consistency (e.g., refining role definitions) while strengthening organizational support systems (e.g., enhancing staffing guarantees, designing playful work, and establishing efficient communication channels) [64–66].

Additionally, the research results can also be used to optimize future nursing education. Considering that the consistency dimension of career identity is the strongest protective factor against nurses’ job burnout, we can integrate a professional value reflection module into nursing curricula. This module adopts case‐driven learning, with cases such as career development paths of nurses with high consistency and methods for resolving professional cognitive conflicts. It helps nursing students establish a reasonable alignment between career expectations and workplace realities, thereby reducing the decline in consistency levels caused by expectation‐reality gaps after graduation.

Perceived stress and physical fatigue are core risk factors triggering burnout, which is consistent with the conclusions of multiple studies [67–69]. Furthermore, this study reveals the interaction between perceived stress and consistency: among nurses with high perceived stress, a low sense of consistency strongly promotes nurses’ job burnout. This may be because excessive work stress offsets the buffering effect of consistency, and this finding provides a new perspective for interpreting the dynamic interaction between risk factors and protective factors in the process of burnout development. For nurses with high perceived stress, a stress and identity integrated intervention (combining stress management training with career identity counseling) can be implemented to leverage the regulatory role of consistency in reducing burnout. For departments with a high incidence of physical fatigue (e.g., emergency departments, ICUs), the workload and recovery experience balance mechanism should be optimized. Examples of such optimization include adding temporary support positions to reduce prolonged high‐intensity work and promoting psychological detachment training to improve rest quality. These measures aim to address fatigue and reduce the risk of job burnout. In addition, consistency, perceived stress, and physical fatigue can be included in the annual mental health assessment indicators for nurses to achieve early identification and intervention of job burnout risks.

Our study only included 1400 female clinical nurses from a specific region. Regional differences may restrict the generalization of conclusions to other regions or international settings, reducing external validity. Additionally, our study used a cross‐sectional design which cannot establish causality.

In the future, multicenter longitudinal studies can be conducted to track the long‐term impact of dynamic changes in factors such as consistency, perceived stress, and physical fatigue on job burnout. Meanwhile, expanding the sample size to include more diverse nurse populations can further enhance the generalizability of the research results.

## 5. Conclusions

We constructed interpretable predictive models using ML and SHAP for predicting job burnout among female nurses and found that XGBoost performed the best. The result confirmed the role of consistency, perceived stress, and physical fatigue as key protective factors, and consistency exhibited interactions with perceived stress, organizational support, psychological detachment, and sense of control to nurses’ job burnout. This helps identify nurses at risk of job burnout and provide targeted strategy to alleviate nurses’ job burnout.

## Disclosure

All authors read and approved the final manuscript. The funder has no role in the study design, data collection, and data analysis and in writing the manuscript.

## Conflicts of Interest

The authors declare no conflicts of interest.

## Author Contributions

Xue Hu contributed to the acquisition and analysis of data, drafting, and the revision of the manuscript. Chong Liu contributed to the acquisition of data, drafting, and the revision of the manuscript. Xiaoshi Yang was responsible for the conception, design, drafting, and the revision of the manuscript.

## Funding

This study was supported by a grant from the Cooperative Project of Economic and Social Development of Liaoning Province (No. 2024lslybhzkt‐31).

## Supporting Information

Scale S1: Detailed descriptions of scales.

Figure S1: The LASSO path and alpha value diagram.

Table S1: The results of tuned hyperparameters.

## Supporting information


**Supporting Information** Additional supporting information can be found online in the Supporting Information section.

## Data Availability

The datasets generated or analyzed during the current study are not publicly available because this study was a minor study of a project which focus on job burnout of the nurse. But the datasets are available from the corresponding author on reasonable request.
