# Supplementary material for: Predicting Job Burnout Among Female Nurses in China With Machine Learning and Shapley Additive Explanations
Source: J Nurs Manag. 2025 Dec 28;2025:2572646. doi: 10.1155/jonm/2572646 (PMC12745184; doi:10.1155/jonm/2572646)
Supplement: Supplementary file 1 — Supporting Information Additional supporting information can be found online in the Supporting Information section. [file JONM-2025-2572646-s001.docx]

Supplementary Materials

Scale S1 Detailed descriptions of scales.

Figure S1 The LASSO path and alpha value diagram.

Table S1 The results of tuned hyper-parameters.

Scale S1 Detailed descriptions of scales

Scales included recovery experience, resilience, depression, empathy, anxiety, sleep quality, perceived stress, fatigue, coping styles, and organizational support.

Recovery experience was evaluated using the Recovery Experience Questionnaire developed by Sonnentag and Fritz[1], which includes four dimensions: psychological detachment, relaxation, control, and mastery, with a total of 16 items.

Psychological resilience among nurses was assessed using the Ego-Resiliency Scale, which consists of 14 items[2]. Higher scores on the scale indicate greater psychological resilience. This scale has been widely used in previous studies in China with a good reliability.

The Generalized Anxiety Disorder Scale (GAD-7) [3]was applied to evaluate the symptom of anxiety. The scale consists of 7 items and the scores for each item range from at 0 (completely not/ no difficulty) to 3 (almost daily/ extremely difficult). A higher score indicated a higher level of anxiety.

Depressive symptoms among nurses were assessed using the Patient Health Questionnaire-9 (PHQ-9)[4]. The scale comprises 9 items, each scored from 0 (almost never) to 3 (nearly every day) based on the frequency of occurrence. Higher scores indicate greater severity of depressive symptoms.

Sleep quality was assessed using short forms from the PROMIS[5], with higher scores indicating poorer sleep quality.

Empathy was assessed using the Jefferson Scale of Physician Empathy (JSPE)[6]. The scale includes three dimensions: perspective adoption, compassionate care, and walking in the patient’s shoes, with a total of 20 items. Higher total scores on the scale indicate higher levels of empathy among the respondents.

Perceived stress was assessed using the Perceived Stress Scale-10 (PSS-10)[7]. The scale consists of 10 items, with higher scores indicating higher levels of perceived stress.

Stress coping styles were assessed using the Brief-COPE[8]. The scale includes three dimensions: problem-focused coping, emotion-focused coping, and avoidant coping, with a total of 28 items. Scores are calculated separately for each dimension, and higher scores in each dimension indicate a higher level of the corresponding coping style.

Perceived organizational support was assessed using the Perceived Organizational Support Scale (POS Scale)[9]. Higher scores indicate a higher level of perceived organizational support.

1. Sonnentag S, Fritz C. The Recovery Experience Questionnaire: Development and validation of a measure for assessing recuperation and unwinding from work. *Journal of Occupational Health Psychology*. 2007;12(3):204-221. doi:10.1037/1076-8998.12.3.204
2. Zhao L, Yang F, Sznajder KK, Zou C, Jia Y, Yang X. Resilience as the mediating factor in the relationship between sleep disturbance and post-stroke depression of stroke patients in China: A Structural equation modeling analysis. *Frontiers in Psychiatry*. 2021;12:625002. doi:10.3389/fpsyt.2021.625002
3. Ma R, Yang F, Zhang L, et al. Resilience mediates the effect of self-efficacy on symptoms of prenatal anxiety among pregnant women: a nationwide smartphone cross-sectional study in China. *BMC Pregnancy and Childbirth*. 2021;21(1):430. doi:10.1186/s12884-021-03911-5
4. Zhao L, Yang F, Sznajder KK, Zou C, Jia Y, Yang X. Resilience as the mediating factor in the relationship between sleep disturbance and post-stroke depression of stroke patients in China: A Structural equation modeling analysis. *Frontiers in Psychiatry*. 2021;12:625002. doi:10.3389/fpsyt.2021.625002
5. Yu L, Buysse DJ, Germain A, et al. Development of short forms from the PROMISTM Sleep Disturbance and Sleep-Related Impairment item banks. *Behavioral Sleep Medicine*. 2012;10(1):6-24. doi:10.1080/15402002.2012.636266
6. Hojat M, Gonnella JS, Nasca TJ, Mangione S, Veloksi JJ, Magee M. The Jefferson scale of physician empathy. *Academic Medicine*. 2002;77(Supplement):S58-S60. doi:10.1097/00001888-200210001-00019
7. Cohen S, Kamarck T, Mermelstein R. A global measure of perceived stress. *Journal of Health and Social Behavior*. 1983;24(4):385. doi:10.2307/2136404
8. Carver CS. You want to measure coping but your protocol’ too long: Consider the brief cope. *International Journal of Behavioral Medicine*. 1997;4(1):92-100. doi:10.1207/s15327558ijbm0401_6
9. Eisenberger R, Huntington R, Hutchison S, Sowa D. Perceived organizational support. *Journal of Applied Psychology*. 1986;71(3):500-507. doi:10.1037/0021-9010.71.3.500

Figure S1 The LASSO path and alpha value diagram. (a) alpha value diagram of LASSO-based regression model; (b) LASSO paths based on the optimal tuning parameter.

(a)


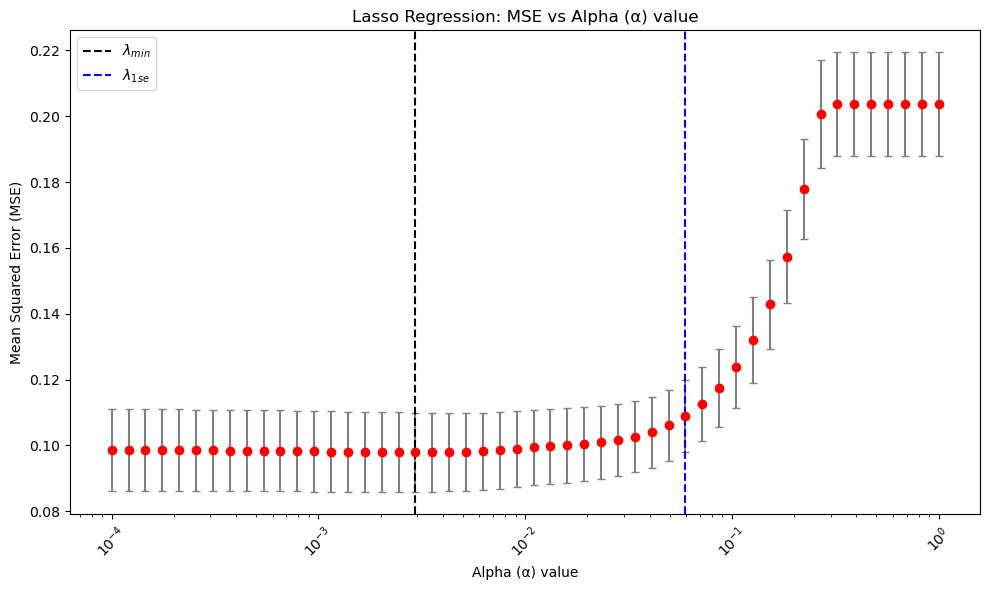


(b)


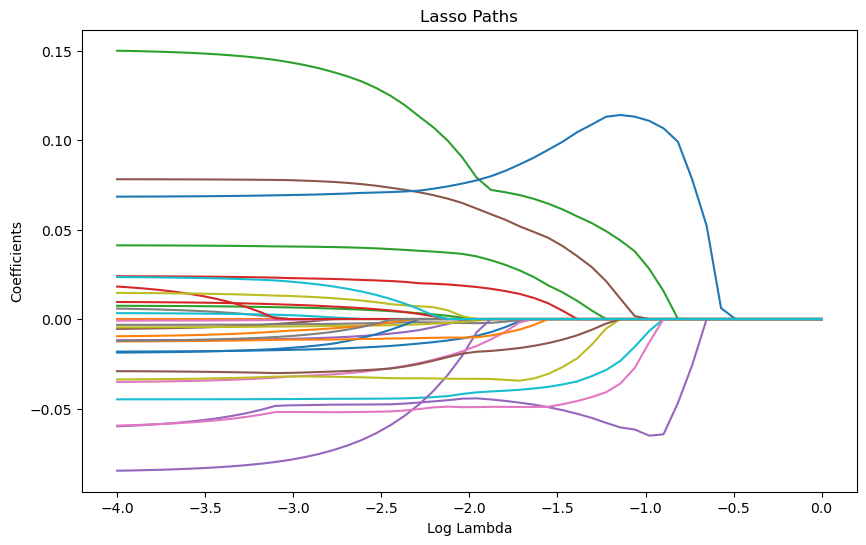


Table S1 The results of tuned hyper-parameters

| ML method | parameters of models | models |
| --- | --- | --- |
| KNN | n_neighbors | 7 |
| RF | Number of estimators | 20 |
|  | Max depth of trees | 8 |
|  | min_samples_leaf | 10 |
|  | min_samples_split | 2 |
| XGBoost | learning_rate | 0.07 |
|  | Number of estimators | 50 |
|  | Gamma (γ) | 0.1 |
|  | reg_lambda | 1 |
